# A Study Conducted on Complications Associated With Percutaneous Nephrostomy (PCN) at a Tertiary Care Center in Peshawar, Pakistan

**DOI:** 10.7759/cureus.80476

**Published:** 2025-03-12

**Authors:** Muhammad Waqas, Asif Khan, Siddiq Akbar, Sulaiman Shah, Shabeer Ahmad, Muhammad Saad Hamid

**Affiliations:** 1 Urology, Institute of Kidney Diseases, Hayatabad Medical Complex, Peshawar, PAK; 2 General Surgery, Hayatabad Medical Complex, Peshawar, PAK

**Keywords:** complications, obstructive uropathy, pakistan, percutaneous nephrostomy, peshawar, success rate, ultrasound-guided pcn

## Abstract

Background

Percutaneous nephrostomy (PCN) is a minimally invasive, image-guided procedure used to relieve urinary obstruction and preserve renal function. However, it carries a risk of both minor and major complications. This study evaluates the indications, success rate, and complications of ultrasound-guided PCN in patients with obstructive uropathy at a tertiary care center in Peshawar, Pakistan.

Methods

A prospective observational study was conducted in the Department of Urology at Hayatabad Medical Complex, Peshawar, over seven months (July 2024 to January 2025) following ethical approval (IREB No. 1886). A total of 117 patients who underwent ultrasound-guided PCN were included. Demographic data, clinical indications, success rates, and complications were documented over a one-week follow-up period. Data were analyzed using IBM SPSS Statistics for Windows, Version 23 (Released 2015; IBM Corp., Armonk, New York, United States).

Results

The mean age of patients was 50.54 ± 11.16 years (range: 27-82), with 68.4% (n = 80) being male and 31.6% (n = 37) female. The most common indication for PCN was malignancy (n = 57, 48.7%), followed by urinary stones (n = 30, 25.6%), pyonephrosis (n = 21, 17.9%), and pelvi-ureteric junction obstruction (n = 9, 7.7%). The overall success rate was 97.4% (114/117). Successful placement was achieved on the first attempt in 13.7% (n = 16) of cases, while 54.7% (n = 64) required two attempts, and 23.1% (n = 27) required three. Minor complications occurred in 29.9% (n = 35) of patients, with catheter blockage (n = 14, 12.0%), hematuria (n = 10, 8.5%), and catheter dislodgement (n = 7, 6.0%) being the most frequent. Major complications were observed in 6.8% (n = 8) of cases, with sepsis (n = 4, 3.4%) and bleeding requiring transfusion (n = 2, 1.7%) being the most common. Hematuria showed a significant association with the number of attempts (p = 0.040), whereas obesity was significantly linked to catheter dislodgment (p = 0.032) and urinary tract infections (UTIs) (p = 0.017).

Conclusion

Ultrasound-guided PCN is a highly effective procedure for managing obstructive uropathy, with a high success rate and relatively low complication rates. The risk of hematuria increases with multiple attempts, while obesity is significantly associated with catheter dislodgment and UTIs. Recognizing these risk factors can help optimize procedural planning and reduce complication rates.

## Introduction

Urinary tract stones, malignancies, and iatrogenic strictures are common causes of obstructive uropathy in adults [1]. Obstructive uropathy, a condition caused by structural blockages in the urinary tract, can lead to hydronephrosis and renal dysfunction. It is responsible for approximately 10% of acute renal failure cases and 4% of end-stage renal disease cases, highlighting its significant impact on kidney health [2]. Percutaneous nephrostomy (PCN), first described by Dr. Willard Goodwin in 1955, is a minimally invasive technique used to relieve urinary obstruction and preserve kidney function [3]. In patients with azotemia due to obstruction, renal function normalizes within 15 days after PCN in two-thirds of cases [4].

Ultrasound-guided PCN is frequently preferred over fluoroscopic guidance due to its lower complication rates and comparable efficacy in treating obstructive uropathy [5]. The procedure involves inserting a tube through the skin into the renal collecting system, allowing urine drainage from the affected kidney [6]. Beyond relieving urinary obstruction in most cases (85%-95%), PCN serves additional purposes, such as providing access for endourological procedures, enabling urinary diversion, and facilitating diagnostic testing [1]. The procedure offers a success rate of 96-100% in dilated collecting systems, 82-96% in non-dilated systems, and 82-85% in complex kidney stone disease [7].

Although PCN is generally safe, it carries inherent risks, and both minor and major complications can occur. According to the quality improvement guidelines for PCN [7], major complications such as septic shock, hemorrhage requiring transfusion, vascular injury requiring embolization or nephrectomy, bowel transgression, and pleural complications are reported in up to 10% of cases. Studies in Pakistan have documented high success rates for PCN, with complication rates ranging from 4.66% to 17.3%, including both minor and major complications [8-11].

Although studies on PCN complications have been conducted in Pakistan, they remain limited, with most originating from the Punjab region. Data from other provinces, particularly Khyber Pakhtunkhwa (KPK), are scarce. Given potential regional differences in patient demographics, procedural expertise, and healthcare resources, this study aims to bridge the gap by evaluating PCN-related complications in a tertiary care center in Peshawar, Pakistan. By providing region-specific data, we seek to improve patient care, procedural safety, and clinical outcomes while contributing to the broader understanding of PCN complications.

Objectives

The objectives of this study are to identify the indications for PCN placement, evaluate its success rate in a tertiary care setting, assess the frequency and types of complications associated with PCN, and determine patient-related risk factors contributing to these complications.

## Materials and methods

Study design and setting

This prospective observational study was conducted in the Department of Urology at Hayatabad Medical Complex (HMC), Peshawar, Pakistan, over a seven-month period from July 2024 to January 2025. Ethical approval was obtained from the Institutional Research and Ethical Board (IREB Approval No. 1886).

Sample size and selection criteria

A total of 117 patients undergoing ultrasound-guided PCN for obstructive uropathy were included. The sample size was calculated using OpenEpi version 3, based on an anticipated septicemia rate of 8.3% [8], a 5% margin of error, and a 95% confidence level. Patients were recruited through non-probability convenient sampling.

Inclusion Criteria

Patients aged 18 years or older who presented with clinical features of obstructive uropathy, such as flank pain, difficulty in urination, dysuria, oliguria, or deranged renal function, with confirmatory radiological evidence (e.g., hydronephrosis or hydroureter) on ultrasonography or computed tomography.

Exclusion Criteria

Patients with an International Normalized Ratio (INR) >1.5, platelet count < 50,000/µL, or those who refused to provide consent were excluded.

Procedure and data collection

All patients underwent a pre-procedure evaluation, including detailed history, clinical examination, baseline laboratory investigations, and ultrasound KUB and CT KUB (Kidney, Ureter, Bladder). Informed consent was obtained after explaining the risks and benefits of the procedure. PCN was performed by post-graduate urology residents and specialist registrars in aseptic condition. Local anesthesia was administered at the puncture site using 5-10 mL of 1% lignocaine, infiltrating up to the muscle layers. PCN was performed using the Seldinger technique [12], involving ultrasound-guided puncture of the pelvicalyceal system, serial tract dilation over a guidewire, and insertion of an 8Fr nephrostomy tube. All patients received preoperative cefoperazone-sulbactam (2g IV) 30-60 minutes before the procedure. Postoperatively, broad-spectrum antibiotics were prescribed to all patients. Patients were monitored post-procedure for complications and were followed up for one week through inpatient monitoring, outpatient clinic visits, and telephone consultations.

Statistical analysis

Data were collected using a structured proforma and analyzed using IBM SPSS Statistics for Windows, Version 23 (Released 2015; IBM Corp., Armonk, New York, United States). Continuous variables were expressed as mean ± standard deviation (SD), while categorical variables were presented as frequencies and percentages. Associations between categorical variables were analyzed using the chi-square test. However, when the expected frequency was less than 5 in more than 20% of the cells, Fisher’s exact test was applied for more accurate results. A p-value < 0.05 was considered statistically significant.

## Results

**Figure 1:**
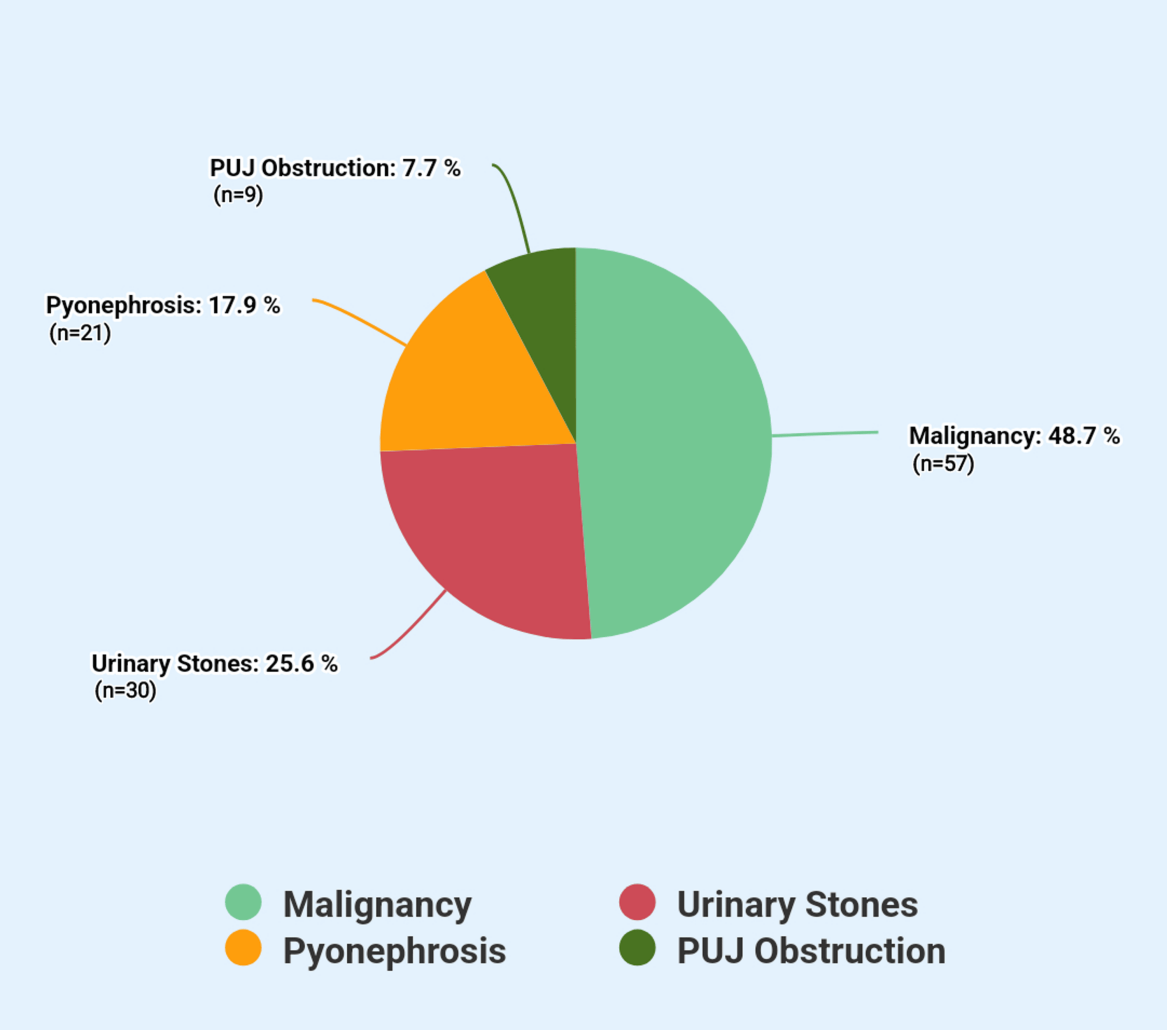
Indications for Percutaneous Nephrostomy in Patients with Obstructive Uropathy PUJ: Pelvi-ureteric junction

A total of 117 patients were included in the study, with an age range of 27 to 82 years (mean: 50.54 ± 11.16 years). The majority were male (n = 80, 68.4%), while female patients were 31.6% (n = 37). Most patients (n = 77, 65.8%) were aged 46 years or older, whereas 40 patients (34.2%) were younger than 45 years. Fifty-one patients (43.6%) underwent right-sided PCN, 34 (29.1%) had left-sided PCN, and 32 (27.3%) underwent bilateral PCN. The most common indication for PCN was malignancy (n = 57, 48.7%), followed by urinary stones (n = 30, 25.6%), pyonephrosis (n = 21, 17.9%), and pelvi-ureteric junction obstruction (n = 9, 7.7%). The distribution of indications is shown in Figure 1.

Hypertension was the most common comorbidity, affecting 31.6% (n = 37) of patients. Diabetes mellitus was present in 29.9% (n = 35), while obesity (BMI ≥ 30) was observed in 20.5% (n = 24) of participants. A total of 35 patients (29.9%) experienced minor complications, with catheter blockage (n= 14, 12.0%) being the most common, followed by hematuria (n = 10, 8.5%) and catheter dislodgment (n = 7, 6.0%). Major complications occurred in eight patients (6.8%), with sepsis (n = 4, 3.4%) being the most frequent, followed by bleeding requiring transfusion (n = 2, 1.7%). Table 1 presents all minor and major complications recorded in the study participants.

**Table 1:** Frequency of Minor and Major Complications Following Percutaneous Nephrostomy UTI: Urinary Tract Infection; GIT: Gastrointestinal Tract; IVC: Inferior Vena Cava

	Complication	Frequency (n)	Percentage (%)
Minor Complications	Catheter Dislodgment	7	6.0
	Catheter Blockage	14	12.0
	UTI	4	3.4
	Hematuria	10	8.5
	Insertion Site Infection	2	1.7
	Urine Leak	5	4.3
Major Complications	Sepsis	4	3.4
	Bleeding Requiring Transfusion	2	1.7
	GIT Puncture	1	0.9
	IVC Puncture	1	0.9

Successful PCN placement on the first attempt was achieved in 16 cases (13.7%), while 64 cases (54.7%) required a second attempt, and 27 cases (23.1%) needed three attempts. For the remaining 10 cases that were unsuccessful after three attempts, PCN was reattempted after 24 hours, leading to seven additional successful procedures (6.0%), while three cases (2.6%) failed and were referred to interventional radiology. The overall technical success rate in this study was 97.4% (114/117 cases). Hematuria was significantly associated with multiple attempts (p = 0.040), suggesting that repeated punctures increase the risk of bleeding, while other complications showed no significant associations (Table 2).

**Table 2:** Association Between the Number of Attempts and Complications Following Percutaneous Nephrostomy UTI: Urinary Tract Infection, GIT: Gastrointestinal Tract, IVC: Inferior Vena Cava

Complications	1st attempt (n=16)	2nd attempt (n=64)	3rd attempt (n=27)	After 24 hours (n=7)	p-value
Catheter Blockage	2 (12.5%)	5 (7.8%)	6 (22.2%)	1 (14.3%)	0.330
Catheter Dislodgment	2 (12.5%)	3 (4.7%)	2 (7.4%)	0 (0%)	0.610
UTI	1 (6.3%)	5 (7.8%)	3 (11.1%)	0 (0%)	0.949
Hematuria	1 (6.3%)	3 (4.7%)	7 (25.9%)	1 (14.3%)	0.040
Insertion Site Infection	2 (12.5%)	1 (1.6%)	0 (0%)	0 (0%)	0.213
Urine Leak	0 (0%)	2 (3.1%)	3 (11.1%)	0 (0%)	0.407
Sepsis	1 (6.3%)	2 (3.1%)	1 (3.7%)	0 (0%)	0.848
Bleeding Requiring Transfusion	0 (0%)	1 (1.6%)	1 (3.7%)	0 (0%)	0.703
GIT Puncture	1 (6.3%)	0 (0%)	0 (0%)	0 (0%)	0.222
IVC Puncture	0 (0%)	1 (1.6%)	0 (0%)	0 (0%)	1.000

Obesity was significantly associated with catheter dislodgment (p = 0.032) and urinary tract infection (UTI) (p = 0.017). No significant associations were found between other complications and comorbidities (Table 3).

**Table 3:** Association Between Patient Comorbidities and Complications UTI: Urinary Tract Infection, GIT: Gastrointestinal Tract, IVC: Inferior Vena Cava

Complications		Hypertension (n=37)	Diabetes Mellitus (n=35)	Obesity (n=24)
Catheter Blockage	N (%)	4 (10.8%)	2 (5.7%)	4 (16.7%)
	p-value	1.000	0.224	0.482
Catheter Dislodgment	N (%)	3 (8.1%)	2 (5.7%)	4 (16.7%)
	p-value	0.677	1.000	0.032
UTI	N (%)	2 (5.4%)	2 (5.7%)	5 (20.8%)
	p-value	0.717	0.723	0.017
Hematuria	N (%)	7 (18.9%)	2 (5.7%)	4 (16.7%)
	p-value	0.50	0.506	0.264
Insertion Site Infection	N (%)	0 (0%)	1 (2.9%)	2 (8.3%)
	p-value	0.551	1.000	0.106
Urine Leak	N (%)	2 (5.4%)	1 (2.9%)	2 (8.3%)
	p-value	0.651	1.000	0.272
Sepsis	N (%)	1 (2.7%)	2 (5.7%)	1 (4.2%)
	p-value	1.000	0.582	1.000
Bleeding Requiring Transfusion	N (%)	0 (0%)	0 (0%)	0 (0%)
	p-value	1.000	1.000	1.000
GIT Puncture	N (%)	0 (0%)	1 (2.9%)	0 (0%)
	p-value	1.000	0.299	1.000
IVC Puncture	N (%)	1 (2.7%)	0 (0%)	0 (0%)
	p-value	0.316	1.000	1.000

The most common complications among male patients were hematuria (n = 10, 12.5%), catheter blockage (n = 9, 11.3%), and UTI (n = 7, 8.8%). Among female patients, the most frequent complications were catheter blockage (n = 5, 13.5%), insertion site infection (n = 2, 5.4%), hematuria (n = 2, 5.4%), and UTI (n = 2, 5.4%). However, no statistically significant association was found between gender and any of the complications (p > 0.05 for all comparisons). Similarly, there were no statistically significant associations between complications and age (p > 0.05 for all comparisons) (Table 4).

**Table 4:** Distribution of Complications Following Percutaneous Nephrostomy by Age and Gender UTI: Urinary Tract Infection, GIT: Gastrointestinal Tract, IVC: Inferior Vena Cava

Complications	Below 45 years of age (n=40)	46 years old and above (n=77)	p-value	Male (n=80)	Female (n=37)	p-value
Catheter Blockage	3 (7.5%)	11 (14.3%)	0.376	9 (11.3%)	5 (13.5%)	0.764
Catheter Dislodgment	2 (5.0%)	5 (6.5%)	1.000	6 (7.5%)	1 (2.7%)	0.429
UTI	3 (7.5%)	6 (7.8%)	1.000	7 (8.8%)	2 (5.4%)	0.717
Hematuria	4 (10.0%)	8 (10.4%)	1.000	10 (12.5%)	2 (5.4%)	0.334
Insertion Site Infection	2 (5.0%)	1 (1.3%)	0.269	1 (1.3%)	2 (5.4%)	0.235
Urine Leak	2 (5.0%)	3 (3.9%)	1.000	4 (5.0)	1 (2.7%)	1.000
Sepsis	1 (2.5%)	3 (3.9%)	1.000	2 (2.5%)	2 (5.4%)	0.590
Bleeding Requiring Transfusion	1 (2.5%)	1 (1.3%)	1.000	2 (2.5%)	0 (0%)	1.000
GIT Puncture	1 (2.5%)	0 (0%)	0.342	0 (0%)	1 (2.7%)	0.316
IVC Puncture	0 (0%)	1 (0.9%)	1.000	1 (1.3%)	0 (0%)	1.000

## Discussion

PCN remains a cornerstone in the management of obstructive uropathy, offering a minimally invasive solution to alleviate renal obstruction and preserve kidney function. This study aimed to evaluate the indications, success rates, and complications associated with ultrasound-guided PCN at Hayatabad Medical Complex, Peshawar, Pakistan.

The technical success rate of PCN placement in our study was 97.4%, aligning with the reported range of 82-100% in the quality improvement guidelines for PCN [7]. Similar success rates have been documented in Bahawalpur (97.5%) [11] and India (95.4%) [13]. However, a lower success rate of 81% has been reported in Ethiopia [14]. In our study, more than half of the procedures (54.7%) were successful on the second attempt, while 23.1% required three attempts, and only 13.7% were completed on the first attempt. In contrast, an Indian study [13] reported a single-attempt success rate of 86.1%, with only 13.9% requiring multiple attempts. No data on the number of attempts is available in previous Pakistani studies [8,11], highlighting the need for further research in this area.

Our study found that most patients were male (68.4%), with no statistically significant association between gender and complications. These findings are consistent with that of the study conducted in Rahim Yar Khan [8], where 57.14% of patients were male, and no association between gender and complications was found (p = 0.472). Similarly, most patients were male in Bahawalpur (72%) [11] and India (58.1%) [13]. A study from Ethiopia [14] reported a predominantly female population (70%).

The mean age of patients in our study was 50.54 ± 11.16 years, with 65.8% of patients aged 46 years and above. No statistically significant association was found between age groups and complications (p > 0.05). Similarly, the Rahim Yar Khan study [8] reported a mean age of 44.83 ± 13.05 years, with 68.42% of patients in the 41-60-year age group and no significant association between age and complications (p = 0.743). This trend was also observed in studies from Ethiopia (mean age: 48 ± 12.9 years), where more than half of the patients were in the 41-60-year age range [14], and India (mean age: 47.3 years) [13]. However, the Bahawalpur study [11] reported a younger patient population (mean age: 40 ± 9.65 years).

Among comorbidities, hypertension (31.6%) was the most prevalent, followed by diabetes mellitus (29.9%) and obesity (20.5%). In an Ethiopian study [14], hypertension and diabetes were present in 45.5% of patients, while 54.5% had no comorbidities. However, previous Pakistani studies [8,11] did not report relevant data on comorbidities.

In our study, malignancy (48.7%) was the most common indication for PCN, consistent with findings from Ethiopian [14] and Indian [13] studies. However, this differs from the Bahawalpur study [11], where urinary stones (65%) were the most frequent cause.

Complications of PCN placement vary across studies. Previously published studies [7,15,16] reported major complications (including adjacent organ injury, severe bleeding requiring transfusion, and sepsis) in up to 10% of cases, with minor complications occurring in up to 28%. Our findings were comparable, with minor complications occurring in 29.9% of patients, the most common being catheter blockage (12.0%), followed by hematuria (8.5%) and catheter dislodgement (6.0%). Major complications were observed in 6.8% of cases, with sepsis (3.4%) and bleeding requiring transfusion (1.7%) being the most frequent. The Bahawalpur study [11] reported a 15% complication rate, with bleeding (4.5%) being the most frequent. In contrast, an Ethiopian study [14] reported a 41.8% overall complication rate, with tube blockage (15%) and dislodgement (11%) being the most common. The Rahim Yar Khan [8] identified septicemia (8.33%) as the leading complication, followed by catheter dislodgment (6%) and bleeding (3%). A prospective randomized study in Egypt [17] found hematuria in 10% of patients undergoing ultrasound-guided PCN, compared to 26% in the fluoroscopy-guided group (p = 0.037). Similarly, a 2016 study by Wang in China [15] reported that only 2% of patients experienced significant blood loss requiring transfusion, comparable to our study (1.7%).

Our study found obesity to be significantly associated with catheter dislodgment (p = 0.032) and urinary tract infection (p = 0.017). However, no statistically significant differences were found between obese and non-obese patients for other complications. These findings were similar to those of the Rahim Yar Khan study [8], where complications such as septicemia, bleeding, and PCN dislodgement occurred in both obese and non-obese groups but showed no statistically significant association with obesity (p = 0.649). The association between hypertension or diabetes and PCN complications has not been assessed in previous Pakistani studies [8,11]. However, our study evaluated this relationship and found no significant association.

Strengths and limitations

The strengths of this study include prospective data collection, which minimizes recall bias and ensures accurate documentation. The use of standardized ultrasound-guided PCN reduced procedural variability. Additionally, the study provides a detailed analysis of both minor and major complications and their association with clinical variables such as number of attempts, comorbidities, and gender, offering valuable insights.

The use of non-probability convenience sampling introduces potential selection bias, which may limit the generalizability of our findings. Additionally, as this study was conducted at a single center, the results may not be representative of broader patient populations. Furthermore, the one-week follow-up period may not have been sufficient to capture late complications. A randomized or multi-center study with a longer follow-up period could enhance the external validity of our findings and provide a more comprehensive assessment of long-term nephrostomy tube outcomes.

## Conclusions

Ultrasound-guided PCN demonstrated a high technical success rate with a relatively low incidence of major complications. The most common minor complication was catheter blockage, while sepsis was the most frequently observed major complication. Hematuria was significantly associated with multiple puncture attempts, highlighting the impact of procedural experience. Additionally, obesity was significantly linked to catheter dislodgment and UTI. These findings underscore the importance of optimizing procedural planning, particularly for high-risk groups such as obese patients. Future multicenter studies with longer follow-up periods are necessary to understand the long-term complications associated with the PCN procedure.
